# Development of a comprehensive risk prediction model for arterial stiffness assessment in individuals with obesity

**DOI:** 10.3389/fmed.2024.1430437

**Published:** 2024-08-19

**Authors:** Denisa Pescari, Andreea Borlea, Simina Mihuta, Dana Stoian

**Affiliations:** ^1^Department of Doctoral Studies, “Victor Babeş” University of Medicine and Pharmacy, Timişoara, Romania; ^2^Center for Molecular Research in Nephrology and Vascular Disease, “Victor Babeş” University of Medicine and Pharmacy, Timişoara, Romania; ^3^Discipline of Endocrinology, Second Department of Internal Medicine, “Victor Babeş” University of Medicine and Pharmacy, Timişoara, Romania

**Keywords:** obesity, overweight, arterial stiffness, pulse wave velocity, bioimpedance

## Abstract

**Introduction:**

Obesity in adults is a known risk factor for cardiovascular events and is associated with a decline in arterial elasticity. This study aims to evaluate the utility of pulse wave analysis (PWA) parameters in routine clinical practice for the primary prevention of cardiovascular events by developing a prediction model for arterial stiffness among obese and overweight individuals.

**Methods:**

The study enrolled 84 adult patients, aged 18 to 85 years, with varying degrees of weight status, including optimal weight, overweight, and obesity. The lifestyle habits, the personal and family history of cardiometabolic diseases, as well the clinical evaluation that included BMI (body mass index), WHR (waist-to-hip ratio), WC (waist circumferance) were performed. PWA evaluation was conducted using the Mobil-O-Graph device, assessing the following parameters: pulse wave velocity (PWV), augmentation index (AIx), heart rate (HR), central pulse pressure (cPP), peripheral and central blood pressure (SBP, DBP, cSBP, cDBP). Body composition analysis was performed using the TANITA BC-418 body analyzer. Laboratory results from the past 3 months were also collected during initial nutritional consultations for each patient.

**Results:**

Family history of cardiovascular events showed positive correlations with all PWA parameters, while diabetes history only with PWV and family history of obesity with PWV, DBP, and cSBP. Insufficient sleep duration showed positive associations with all arterial stiffness parameters except cDBP. Smoking status correlated with significantly elevated PWV and Aix values, while insufficient physical activity was associated solely with PWV. Positive correlations were showed between current weight and PWV, while WC demonstrated positive associations with PWV, SBP, and cSBP. Body composition analysis revealed significant associations between trunk adipose tissue mass (%) and PWV, SBP, and cSBP. Hydration status (%) emerged as an independent predictor for PWV, exhibiting an inverse relationship. HOMA-IR (Homeostatic Model Assessment for Insulin Resistance) showed a strong positive correlation with PWV. Negative associations were observed with HDL-c and vitamin D. Threshold values for age, cDBP and Cardiac Index providing positive diagnostic for vascular impairment.

**Conclusion:**

The assessment of arterial stiffness can be considered a reliable approach to prevent obesity-related cardiovascular events and facilitate the comprehensive management of such pathologies.

## 1 Introduction

Obesity represents a complex and insidious pathology characterized by multiple associated complications. Its prevalence is continuously increasing worldwide, thus classifying it as a significant public health concern. Environmental variables including dietary habits, physical activity, and exposure to environmental pollutants are major contributors to the increasing incidence of obesity, even though genetic and epigenetic factors are well-established causes of it (1). According to World Health Organization and the US Center for Disease Control, in 2016, 13% of adults over the age of 18 were obese and 39% were overweight (2, 3). Excess weight has been linked with cardiovascular diseases, insulin resistance, but also with the general increase in morbidity and mortality (4). As a result, a high BMI (body mass index) has been responsible for approximately 60% of deaths globally (5). The associated comorbidities depend on the evolutionary history of obesity, blood vessel damage representing one of the complications with an unfavorable long-term prognosis (6). The key risk factors for coronary and cerebrovascular disease in adults are well defined: excessive inadequate nutrition, unhealthy lifestyle, sedentary lifestyle, harmful alcohol consumption and smoking (7). Excess adipose tissue, arterial hypertension, changes in glycemic values, and lipid profile, but also chronic inflammatory syndrome have been identified among patients presenting these dietary patterns (8). Other diseases that can possibly influence arterial stiffness are thyroid and autoimmune diseases (9).

Considering the aggravating environmental factors and the genetic predisposition for cardiometabolic pathology, the presence of older adults, regardless of body weight, can cause pathological changes in the parameters evaluated in the measurement of arterial stiffness and arterial tension, but obesity is an additional factor for amplifying cardiovascular risk. Therefore, the highlighted values are correlated with the anthropometric parameters, the parameters associated with the electrical bioimpedance but also the biological ones, determining a broad picture of the cardiovascular condition associated with overweight and obese adults compared to normal-weight adults. Women are more likely than males to experience the progressive rise in vascular stiffness linked to obesity; this is because women with insulin resistance lose the cardiovascular protective effects of estrogen (10, 11). Considering the constant increase in the number of cardiovascular events worldwide, one of the effective methods for their prevention is the early documentation of vascular disorders. Atherosclerosis and implicitly the formation of atheroma plaques are processes that lead over time to arterial stiffness, vascular dysfunction, and hypertension, both in children and in young adults (12).

Associated, obesity constitutes the aggravating factor of these pathological mechanisms, leading to or accelerating the possible complications. Especially adipose tissue in the abdominal region has been identified as a risk factor for atherosclerosis, respectively for the loss of arterial elasticity (10, 13, 14). The earliest sign associated with cardiovascular damage secondary to obesity is the dysfunction of relaxation mediated by nitric oxide (NO), which will have the main consequence of arterial stiffness (1). This NO damage is attributed to the inflammatory syndrome, but before that, arterial elasticity is primarily influenced by age, which is an unmodifiable factor with the greatest impact on vascular dysfunction (15, 16). Among the general population, screening to reduce global cardiovascular risk through the early detection of subclinical atherosclerosis would represent a challenge for clinicians (17, 18). The structural and functional remodeling of the arterial wall is the consequence of arterial stiffness, an early subclinical parameter of vascular aging (18–20).

From a clinical perspective, pulse wave velocity measurement (PWV), which is the most often used non-invasive technique for assessing arterial stiffness in adults, is regarded as the gold standard (21, 22). PWV is defined as the pressure waves that are propagated throughout the artery tree by the heart's systolic contraction (23). Vascular elastic characteristics are evaluated by PWV by means of pulse wave travel speed at the level of the arterial tree (24, 25). On the other hand, the significant cardiovascular prognostic value, accuracy, and cost-effectiveness of PWV are acknowledged in current European guidance (26). An increase in PWV value by 1.0 m/s has been associated with an increased risk of mortality from any cause ranging between 6%−15%, the occurrence of cardiovascular events by 12%−14%, and mortality from cardiovascular causes by 13%−15% (27–29). In adults, organ damage and cardiovascular events can be predicted using the wave reflection parameter known as the Augmentation Index (AIx) (30, 31). Additional information on the degree of elasticity at the level of small arteries are provided by this indirect marker that detects arterial stiffness (7, 31).

In the general population, the presence of the atherosclerosis process in the coronary arteries and aortic arterial stiffness are established markers for subclinical vascular dysfunction (32). At the same time, among people with type 2 diabetes, it was found that there is an association between arterial stiffness and this pathology, with an increased incidence of type 2 diabetes cases associated with vascular dysfunction being reported (33). Although a causal relationship between type 2 diabetes and arterial stiffness has not been identified, an additional risk of type 2 diabetes has been associated with both carotid and aortic stiffness (34, 35). Among both type 2 diabetes and prediabetes patients, an increase in PWV was observed compared to people with optimal glycemic values (36, 37). The increase in arterial stiffness is an independent predictor of insulin resistance, respectively of type 2 diabetes in the general population (38). In addition to having a greater impact on aortic stiffness than carotid stiffness (39), aging is a major risk factor for type 2 diabetes and cardiovascular disorders (19). Nevertheless, other data clearly confirms that age had no effect on the relationship between type 2 diabetes and arterial stiffness measurements (34).

Various devices have been developed and validated to assess PWV, employing methods such as applanation tonometry, cuff oscillometry, photodiode sensors, piezoelectric mechano-transducers, ultrasound, and magnetic resonance imaging. However, the most commonly used methods in studies are applanation tonometry and cuff oscillometry. PWV measurement typically involves detecting the transcutaneous pulse wave at two distinct locations on the arterial tree or estimating it by analyzing the impact on the waveform of the reflected wave (6). Numerous studies have been conducted to identify an optimal value for this parameter; however, variations exist depending on various factors, with age and geographical area being the most common influencing factors. Despite ongoing efforts, uncertainties persist in determining an optimal PWV threshold. Díaz et al. established an average PWV value of 6.84 m/s ± 1.65 in a cohort of 780 individuals of varying ages from Argentina, devoid of associated cardiometabolic pathologies such as diabetes, hypertension, or dyslipidemia, and spanning rural and urban areas (40). The 2007 ESH/ESC guideline for hypertension proposed a fixed threshold value of 12 m/s, overlooking additional factors influencing PWV values (41). Inuzuka et al. highlighted that a PWV value of 8.2 m/s exhibited sensitivity in the early assessment of cardiovascular biomarkers indicative of target organ damage (42).

This prospective observational study aims to evaluate the usefulness of determining arterial stiffness markers in assessing and measuring the extent of vascular dysfunction with the aim of obtaining a prediction model of risk factors for endothelial damage among individuals with overweight and obesity who present additional cardiometabolic risk factors. Adding the assessment of arterial stiffness as a screening method among obese patients can be useful for leading a better management and effective prevention of cardiovascular events.

## 2 Materials and methods

The observational prospective study was performed in our endocrinologic unit starting in June 2023 until December 2023. The study group comprised 84 adults, willing to start a personalized lifestyle changing plan, in order to lose weight, mean age 41 ± 17.71, range 18–85 years, 17 females (20.48%) and 67 males (79.51%).

Depending on the severity of excess weight, three study groups were divided, respectively normal weight (BMI between 18–24.9 kg/m^2^), overweight (BMI between 25–29.9 kg/m^2^), and obese (BMI over 30 kg/m^2^). Patients with overweight, respectively primary obesity constituted the study group. The control group included normal weight people, without additional risk factors and without family history of cardiovascular and metabolic diseases, non-smokers. Subsequently, subgroups with smoking and non-smoking patients and also both people with type 2 diabetes and prediabetes were described.

### 2.1 Patient inclusion and exclusion criteria

**Inclusion criteria:** patients regardless gender, overweight and obese, over 18 years of age, who willingly addressed the nutritional counseling services to implement an individualized hypocaloric dietary plan, with a positive family history of cardiovascular and metabolic diseases, smokers and non-smokers, and the control group, people with BMI between 18–24.9 kg/m^2^, without significant vascular and metabolic hereditary collateral antecedents. Only patients willing to complete the entire evaluation and signing the informed consent were included in the final evaluation.

**Exclusion criteria:** patients with documented psychiatric pathology, patients who have followed a hypocaloric food plan in the last 12 months or previous users medication for weight loss in the last 16 weeks (semaglutide, liraglutide, Orlistat, and Bupropion/Naltrexone) (43). Patients diagnosed with secondary obesity, regardless etiology: iatrogenic—insulin therapy, glucocorticoids, antipsychotics, genetic endocrinologic—Cushing's syndrome, hypothyroidism, hypogonadism, or genetic—Prader-Willi syndrome, were excluded from the research (43–45).

### 2.2 Patient evaluation

Before performing any procedure, the patient was familiarized with the details of the study, clinical and paraclinical needed examinations. Each patient was given informed consent to be accepted and signed. The primary non-invasive methods used in our investigation were the oscillometric evaluation assessing the arterial stiffness parameters, respectively bioimpedance measurements in order to estimate the segmental body composition.

A targeted anamnesis was performed, in order to highlight the potential cardiovascular risk factors, such as: dietary routine, cardiometabolic personal medical history (prediabetes, type 2 diabetes, manifest clinical hypothyroidism, documented cardiovascular disease, metabolic syndrome, hyperuricemia, and dyslipidemia), as well as the family medical history (data of interest are represented by first-degree relatives known to be obese and overweight, documented cardiovascular diseases, including hypertension, stroke, acute myocardial infarction, type 2 diabetes, and prediabetes). At the same time, the complete clinical examination was performed in each patient. The evaluation at study begin included the following parameters:

- **Height**: the height of each patient was assessed by means of a mounted calibrated tally meter, thus the vertical posture on the platform was measured without wearing any shoes.
- **Body weight:** The body weight of each participant at the time of the presentation was assessed using a metrologically certified mechanical scale, whose maximum allowed weight is 180 kg, with the basic procedure being explained to each subject, namely maintaining a vertical posture on the concomitant device with minimal clothing.
- **Nutritional status:** nutritional status consisted of estimating BMI, a cheap, frequently used and inexpensive parameter, among each subject in the study as a result of estimating the previous parameters, using the following calculation formula: BMI = weight (in kg)/height^2^ (in m^2^) (46, 47).
- **Cigarette smoking status:** Smoking at least one cigarette every single day for more than a year was considered cigarette smoking status.
- **Physical activity level:** A minimum of 30 min each day or 150 min per week (activity level > active plus basal) was considered essential for someone to not be classified as sedentary.
- **Alcohol consumption:** To quantify alcohol consumption, participants were required to report the number of units of alcohol consumed (equivalent to 10 ml of pure ethanol) through self-reporting. These units were defined as follows: two units corresponded to a pint or can of beer, one unit to a single shot of 25 ml of hard liquor, and to a standard glass of white or red wine (175 ml). Participants who reported consuming more than two units of alcohol daily were categorized as “alcoholic,” while those who had never consumed alcohol were classified as “non-alcoholic” (48).

The following biological data were assessed and collected at the initial moment of the study: fasting glycemia (mg/dl), lipid panel: total cholesterol-TC (mg/dl), low-density lipoprotein (LDL-c) (mg/dl), high-density lipoprotein-HDL-c (mg/dL), triglycerides-TG (mg/dl), uric acid (mg/dl), glycated hemoglobine-HbA1c (%), thyroid stimulating hormone (TSH), free thyroxine (FT4), homeostasis model assessment-estimated insulin resistance (HOMA-IR), triglyceride-glucose index-TyG index, and 25-OH-Vitamin D (ng/mL). The following formula was used to generate the TyG index: [fasting triglycerides (mg/dL) × fasting glucose (mg/dL)/2] (49).

### 2.3 Assessment of arterial stiffness using the Mobil-O-Graph

The oscillometric device Mobil-O-Graph^®^ 24 h ABPM (M26101200, IEM^®^ GmbH, Stolberg, Germany) with Hypertension Management Software CS (IEM GmbH, Aachen, Germany) was used. The Mobil-O-Graph IEM is a validated tool, using the SphygmoCor device as standard (50). It is considered a simple and reproducible technique, non-invasive and without associated risks, useful for both adults and children (50, 51).

M26101200 directly measures both peripheral blood pressure at unilateral brachial level: systolic (SBP) and diastolic (DBP), and secondly, after 30 second free interval, performs the pulse wave analysis, with the following determined parameters: pulse wave velocity (PWV), mean arterial pressure (MAP), augmentation index (AIx), central systolic blood pressure (cSBP), central diastolic blood pressure (cDBP) and heart rate (HR).

The oscillometric measurement was performed for each patient at the initial evaluation before of any nutritional intervention. The technique was explained to each patient, as well as the protocol to be followed: avoiding the consumption of liquids or foods with added caffeine and smoking at least 4 h before the procedure, presence of a restful sleep by 10 pm the night before the assessment. The 10 min resting, in supine position, before the oscillometric evaluation was applied to each patient. For each subject, the correct size of the cuff of the secondary arm determination device was used (32–38 cm-L size, 24–32 cm-M size, 20–24 cm- S size, 14–20 cm- XS size). The appropriately chosen cuff was placed with the sign of the artery at the level of the brachial artery and with the pressure tube toward the apex. The patients were advice to be still and silent during the measurement (52). At the end of the procedure, the device will accept or reject the measurement for increased accuracy. Incorrectly performed measurements will be repeated after a 300 second pause (52).

### 2.4 Bioimpedance body analyzed parameters

The TANITA BC-418 segmental body composition analyzer model was used for the body composition measurement, a device that certifies a valid measurement of body composition using segmental analysis compared to DXA (53, 54). The participants were taught to keep a straight, vertical position, not moving during the procedure, to touch the handles of the device to create a connection between the eight electrodes (2 each for both the upper and lower ones) (55). The device is based on the principle of impedance determination using a low-level electric flux (56). Thus, the parameters evaluated by this technique were classified into: adipose tissue (%), trunk adipose mass (%), fat free mas (kg), muscle tissue (%), metabolic basal rate (RMB), and body water (%).

### 2.5 Personal and family medical history

- Familial cardiometabolic risk factors: There was evaluated the presence of following metabolic and cardiovascular pathologies in the 1st degree relatives: diabetes type 2, abdominal obesity, essential hypertension, ischemic or hemorrhagic stroke, heart failure, peripheral arterial disease, mixed ischemic, and hypertensive heart disease.
- Personal cardiometabolic risk factors: There were recorded the previous mentioned preexistent conditions.
- Lifestyle personal parameters: The following parameters were enquired: <7 h per night, smoking at least one cigarette every single day for more than a year was considered cigarette smoking status, daily alcohol consumption, and less physical activity than 30 min per day or 150 min per week, were considered high risk behavior characteristics.

### 2.6 Statistical analysis

We conducted a thorough statistical analysis to summarize the study population's characteristics. Continuous variables were reported using different measures based on their distribution. Normally distributed data (Gaussian) was presented as mean standard deviation (SD), and for non-normally distributed data, median and interquartile range (Q25–Q75) were utilized. Categorical variables were summarized using frequencies and proportions. We assessed the normality of continuous variable distributions using the Shapiro-Wilk test, considering a *p*-value > 0.05 indicative of a Gaussian distribution. To compare significant differences between two study groups, we employed the Mann-Whitney U test and more than two groups, the Kruskal-Wallis test was conducted. Pairwise comparisons were performed using the Dunn test with Holm's method for *p*-value adjustment. The rank-biserial correlation coefficient was used to evaluate the magnitude of differences between two groups, while the ordinal coefficient (ε^2^) was used for comparisons involving more than two groups. Pearson's test was used to assess significant differences among categorical variables, and Cramer's was employed to quantify the magnitude of these differences. To examine the association between numerical variables, correlation analysis was conducted using Spearman's correlation coefficient, with a coefficient >0.5 considered indicative of a strong correlation. To identify independent predictors for vascular rigidity parameters, both simple and multiple linear regression analyses were performed. Logistic regression analysis was used to identify independent risk and protective factors. Model selection was guided by the Akaike Information Criterion (AIC), with the best model exhibiting the smallest AIC considered optimal. Model evaluation included metrics such as R^2^, R^2^ adjusted, Nagelkerke's R^2^, accuracy, specificity, and sensitivity. Performance assessment utilized ROC curve analysis to determine threshold values for independent risk factors, with the cutoff point optimizing sensitivity and specificity identified using the Youden index. Confidence intervals for the area under the ROC curve (AUC) were calculated using the robust DeLong method.

Data collection, processing, and analysis were carried out with the R software (57). Results were presented in tabular and graphical form. Statistical significance was determined using a significance level of *p*-value < 0.05, with a 95% confidence interval.

## 3 Results

### 3.1 Statistics of investigated variables in the study

Presence of high risk lifestyle habits were recorded as following: smoking (67.5%), alcohol consumption (83.1%), insufficient sleep (67.5%), and sedentarism (73.5%). A noteworthy 62.7% of the study group had a positive family medical history of obesity (FHO), while a significant respectively 71.1% of cardiovascular diseases (FHC), with an even distribution of family diabetes history (FHD), of 50.6%. Personal history of diabetes (PHD) was recorded in twenty-five patients (30.1%).

**Figure 1:**
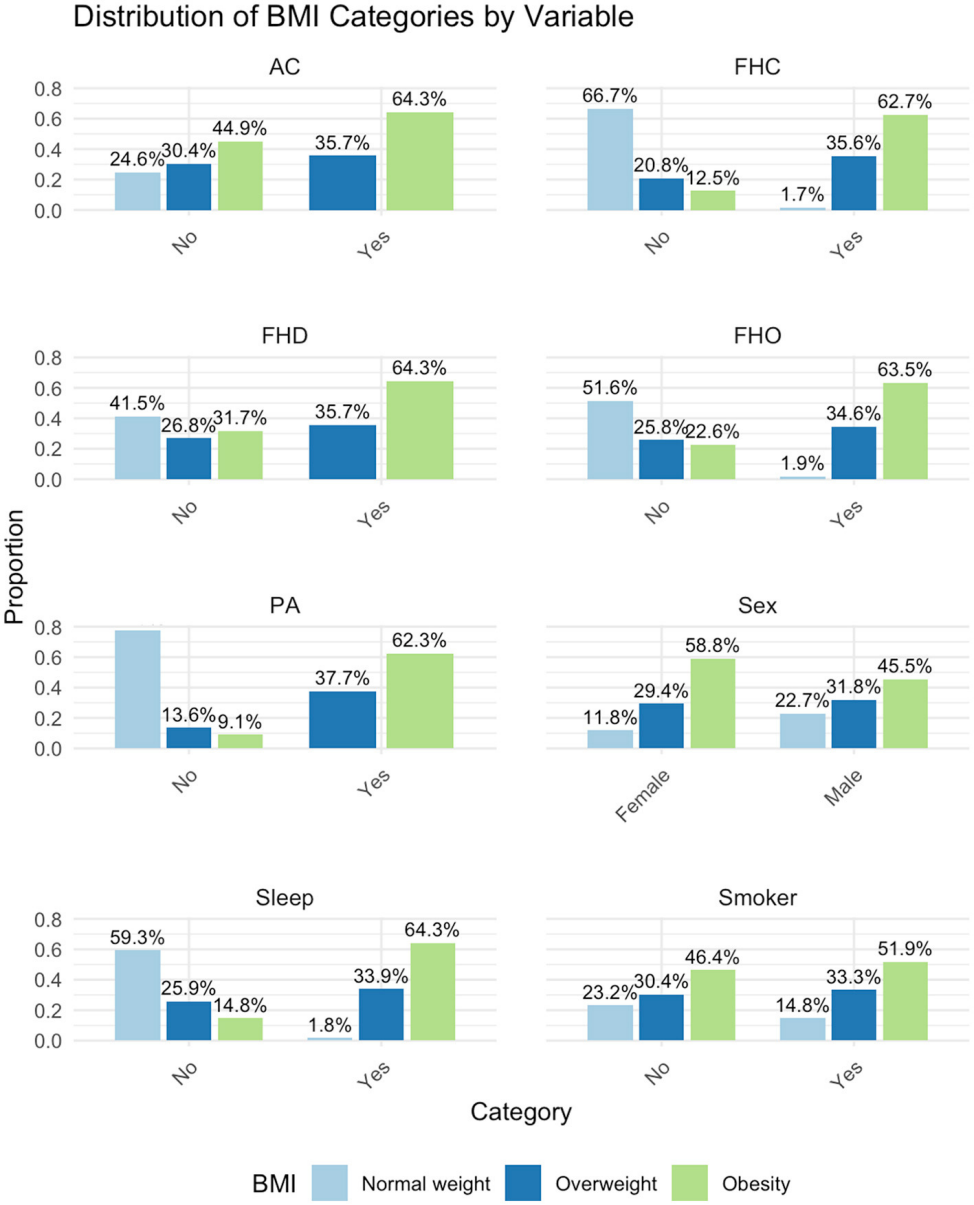
Distribution of BMI categories by variable. BMI, Body Mass Index; FHO, Family Medical History of Obesity; FHD, Family Medical History of Diabetes; FHC, Family Medical History of Cardiovascular Diseases; AC, Alcohol Consumption; PA, Physical Activity (<150 min/week); Sleep, Sleep (<7 h/night).

According to the value of the BMI we divided the study group in 3 subgroups as follows: the control group (normal weight), the overweight and the obesity subgroup. The categorical data related to each subgroup are briefly presented in Table 1 and Figure 1. The statistical significance of differences among these subgroups were assessed using Pearson's Chi-Squared test, as indicated by the *p*-values. Significant differences among the subgroups were observed in FHO, FHD, FHC, duration of sleep, physical activity, and personal history of diabetes mellitus.

**Table 1:** Categorical variables within the study.

**Variable**	**Classes**	**Control group**	**Overweight group**	**Obesity group**	***p-*value**
Sex	Male	15	21	30	0.52
	Female	2	5	10	
Smoker	Yes	13	17	26	0.67
	No	4	9	14	
FHO	Yes	1	18	33	**< 0.001**
	No	16	8	7	
FHD	Yes	0	15	27	**< 0.001**
	No	17	11	13	
FHC	Yes	1	21	37	**< 0.001**
	No	16	5	3	
Alcohol consumption	Yes	17	21	31	0.11
	No	0	5	9	
Sleep (< 7 h/night)	Yes	1	19	36	**< 0.001**
	No	16	7	4	
Physical activity (< 150 min/week)	Yes	0	23	38	**< 0.001**
	No	17	3	2	
Diabetic	Yes	1	6	18	**0.008**
	No	16	20	22	

BMI, Body Mass Index; FHO, Family Medical History of Obesity; FHD, Family Medical History of Diabetes; FHC, Family Medical History of Cardiovascular Diseases. *p*-value, results of Pearson's Chi Squared test. The bold values *p* < 0.05 indicates statistical significance.

Supplementary Table 1 is presenting the anthropometric, cardiovascular assessment, body impedance and lab assays. Anthropometric, lab assays and cardiovascular assessment evaluated variables show a normal distribution: height, weight, BMI, and WC with mean values of 72.98, 96.57, and 75.60, respectively; 72.98, 96.57, and 75.60, respectively: TC, LDL-c, uric acid, and FT4 with mean values of 183.70, 123.80, 5.42, and 10.86, respectively; DBP, MAP, and cDBP with mean values of 72.98, 96.57, and 75.60, respectively. All variables in the bioimpedance tests category demonstrate a non-Gaussian distribution.

There was no significant difference in age and height across the groups. However, significant differences, were observed in weight, WC, and WHR with all parameters increasing from the normal weight to the obesity group, indicating typical anthropometric changes associated with increasing body weight.

Most cardiovascular parameters showed significant differences across weight categories. SBP, DBP, MAP, cSBP, cDBP, PWV, and AIx all increased with higher weight categories. HR and cPP did not show significant differences, indicating some cardiovascular measures were less impacted by weight status.

As for the bioimpedance tests, all measured variables showed significant differences. Fat mass, trunk fat, and body water percentage increased with higher body weight, while fat-free mass and muscle mass percentage decreased. BMR and electrical impedance varied significantly, reflecting changes in body composition with different weight statuses. Significant differences were noted in fasting glucose, HbA1c, HOMA-IR, total cholesterol, LDL-c, HDL-c, triglycerides, 25-OH-Vitamin D, TyG index, and TSH, all worsening with increasing weight. Notably, uric acid levels and FT4 showed no significant difference across the groups. These results indicate worsening metabolic and hormonal profiles as body weight increases.

The data demonstrates significant deteriorations in anthropometric, cardiovascular, bioimpedance, and metabolic parameters as individuals progress from normal weight to overweight and obesity, highlighting the broad systemic impact of increased body fat.

Supplementary Table 1 summarizes the results divided into three groups based on their BMI value: control group (normal weight), overweight group, and obesity group. It presents data comparing these groups using either ANOVA for normally distributed variables (marked with an asterisk) or Kruskal-Wallis tests for non-normally distributed variables.

### 3.2 Exploring variances in vascular stiffness across variables

We analyzed differences among various groups regarding the values of parameters measuring vascular stiffness. There parameters include: PWV, AIx, cPP, cDBP, cSBP, MAP, DBP, and SBP.

We observed that concerning patients' gender, there were no statistically significant differences in parameters, similar to alcohol consumption. Regarding FHC, we found differences in all parameters characterizing vascular stiffness, with the positive FHC group exhibiting higher values for all parameters. In the case of sleep duration, statistically significant differences were observed in all variables except cDBP, with the group sleeping < 7 h showing significant higher values than the group sleeping more than 7 h. Similarly, in the case of physical activity, patients engaging in physical activity for more than 150 min per week obtained better values for vascular stiffness parameters, and this difference was statistically significant. Regarding FHO, patients with a history of obesity presented significantly higher values only in PWV, cSBP, and DBP. In the case of FHD, higher values were observed only in PWV. Smokers exhibited significantly higher values only in PWV and AIx. In terms of BMI, the obesity group obtained significantly higher values in vascular stiffness parameters compared to both the overweight and normal weight groups. We observed that, concerning the diabetic status of patients, those with diabetes exhibited significantly higher values in terms of scores obtained in vascular stiffness measurements, with the most notable being PWV, followed by SBP, AIx, MAP, and cSBP.

The overweight group described significantly higher values than the controls, for all variables describing vascular stiffness, except for cPP. Among the variables characterizing vascular stiffness, we observed that PWV showed statistically significant differences in almost all subgroups except for gender and alcohol consumption, where no statistically significant differences existed for any parameters. The results are summarized in Table 2.

**Table 2:** Differences between groups regarding vascular stiffness parameters.

**Variable**	**PWV**	**AIx**	**cPP**	**cDBP**	**cSBP**	**MAP**	**DBP**	**SBP**
Sex	0.28	0.41	0.21	0.87	0.30	0.60	0.92	0.82
^*^BMI	**< 0.001**	**< 0.001**	0.17	**0.007**	**< 0.001**	**< 0.001**	**0.004**	**< 0.001**
Smoker	**0.03**	**0.02**	0.20	0.21	0.07	0.26	0.16	0.45
FHO	**< 0.001**	0.05	0.40	0.81	**0.04**	0.06	**0.02**	0.65
FHD	**0.03**	0.07	0.43	0.71	0.19	0.09	0.38	0.06
FHC	**< 0.001**	**0.004**	**0.02**	**0.005**	**< 0.001**	**< 0.001**	**0.002**	**< 0.001**
Alcohol consumption	0.83	0.59	0.73	0.24	0.43	0.41	0.45	0.69
Sleep (< 7 h/night)	**< 0.001**	**< 0.001**	**0.03**	0.12	**< 0.001**	**< 0.001**	**0.02**	**< 0.001**
Physical activity (< 150 min/week)	**< 0.001**	**0.001**	0.38	**0.002**	**< 0.001**	**< 0.001**	**0.001**	**< 0.001**
PHD	**< 0.001**	**0.02**	0.17	0.13	**0.04**	**0.03**	0.05	**0.01**

BMI, Body Mass Index; FHO, Family Medical History of Obesity; FHD, Family Medical History of Diabetes; FHC, Family Medical History of Cardiovascular Diseases; SBP, Systolic Blood Pressure; DBP, Diastolic Blood Pressure; MAP, Mean Arterial Pressure; cSBP, Central Systolic Blood Pressure; cDBP, Central Diastolic Blood Pressure; cPP, Central Pulse Pressure; PWV, Pulse Wave Velocity; Aix, Aortic Augmentation Index. Values represent the *p*-values derived from Mann-Whitney U test; ^*^the computed *p*-values from Kruskal-Wallis test. The bold values *p* < 0.05 indicates statistical significance.

Based on Pulse Wave Velocity (PWV) values, regardless weight or gender, we divided the patients into two categories: with vascular impairment (VIG) (PWV > 8) and without vascular impairment (PWV ≤ 8) (NVIG) (40, 42). Subsequently, we explored differences between these two groups concerning the variables included in the study.

As seen in Table 3, the VIG showed significantly higher values for age and BMI compared with the NVIG (Cramer's V = 0.32). Presence of diabetes was significantly higher in the VIG vs. NIVG 46% vs. 17% (Cramer's V = 0.29). In terms of BMI, the proportion of individuals with normal weight is the lowest in the vascular impairment group. Both the proportion of overweight patients and the proportion of obese patients are higher in the vascular impairment group (43% vs. 22% and 51% vs. 46%, respectively), while the proportion of normal weight patients is higher in the non-impairment group (33% vs. 5%). These differences are statistically significant and have a substantial effect size (Cramer's V = 0.32). In terms of the diabetic status of patients, the proportion of patients with diabetes is higher among those with vascular impairment (46% vs. 17%). This difference is statistically significant (*p*-value = 0.004) with a substantial effect size (Cramer's V = 0.29).

**Table 3:** Exploring variances in vascular impairment in the study groups.

**Variables**	**Pearson Chi-squared**	**Cramer's V**
Sex	0.44	0.00
Smoker	**0.02**	0.23
FHO	**0.007**	0.27
FHD	0.15	0.11
FHC	**0.005**	0.29
Alcohol consumption	0.89	0.00
Sleep < 7 h/night	**0.004**	0.29
Physical activity < 150 min/week	**0.003**	0.30
BMI	**0.003**	0.32
Diabetic	**0.004**	0.29

BMI, Body Mass Index; FHO, Family Medical History of Obesity; FHD, Family Medical History of Diabetes; FHC, Family Medical History of Cardiovascular Diseases. Cramer's V, measures the magnitude of the difference with Pearson's X^2^ test. The bold values *p* < 0.05 indicates statistical significance.

For numerical variables, a highly statistically significant difference was observed (*p*-value < 0.001) in age, with a substantial effect size (r biserial = −0.61). The VIG exhibited significantly higher values compared to the NVIG (61 years vs. 33 years median age values). In terms of WHR, the VIG showed significantly higher values (*p*-value = 0.02) compared to the NIVG (0.92 vs. 0.88 median values), with a moderate effect size (r biserial = −0.31). Paradoxically, the VIG had significant lower heart rates (73 b/min vs. 77 b/min, *p*-value = 0.01), with a substantial effect size (r biserial = 0.32).

**Figure 2:**
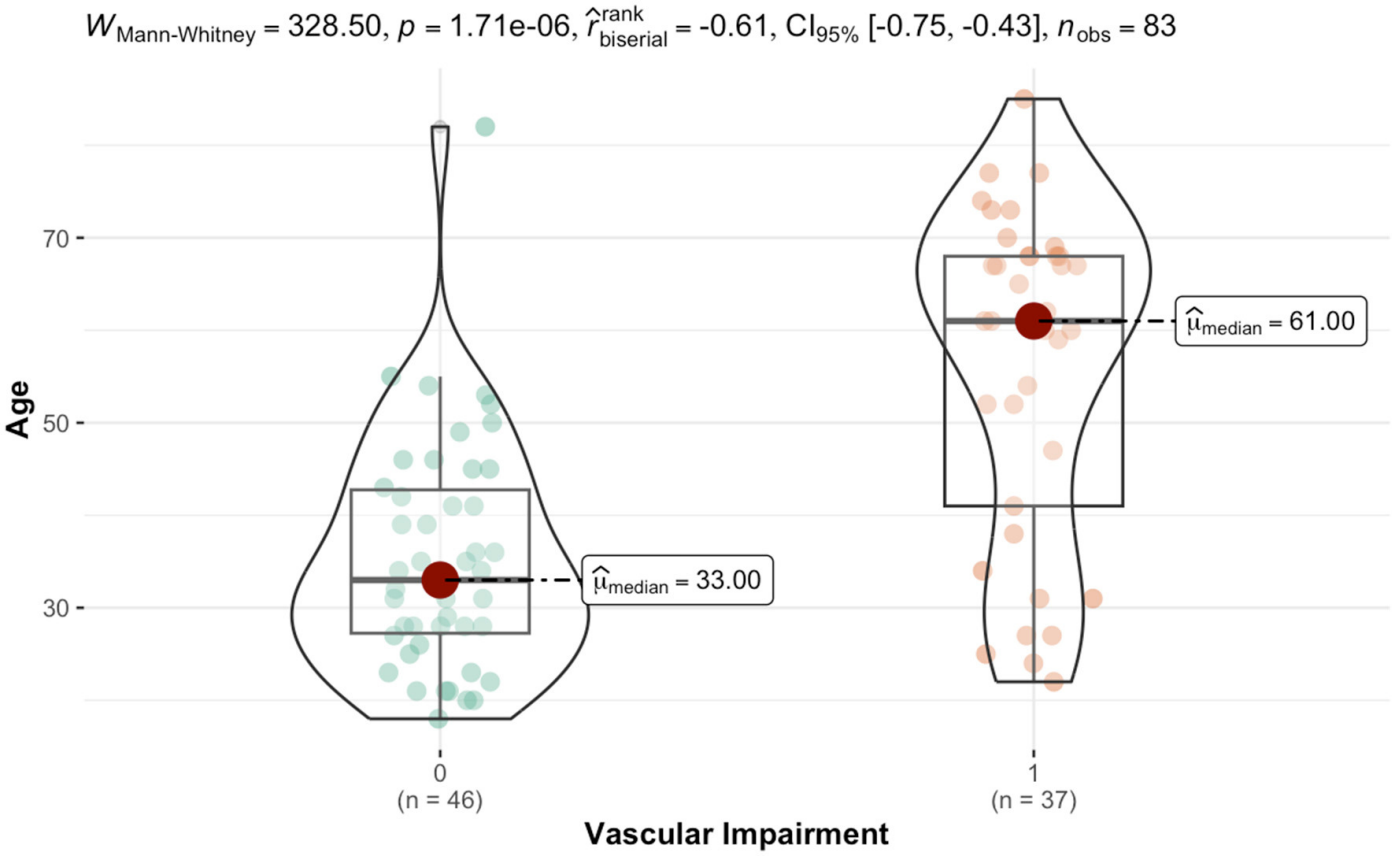
Comparison of age distribution between vascular impairment and non-impairment groups.

The VIG exhibited statistically significant higher values for cardiac index (3.00 L/min/m^2^ vs. 2.50 L/min/m^2^ median values) compared to the NIVG with a substantial effect size (r biserial = −0.41). Fat mass, trunk fat proportion and impedance were significantly higher in the VIG vs. NVIG: 38% vs. 37.15%, (*p*-value = 0.02); 39.30% vs. 37.00% median values (*p*-value = 0.04); 636 Ω vs. 558 Ω median values, (*p*-value = 0.01) all with moderate effect size (r biserial = 0.33), as seen in **Table 6**, Figure 2.

When looking to the lab assays, fasting blood glucose, HbA1c, HOMA-IR, and LDL-c were significantly higher in VIG vs. NVIG: 99 mg/dl vs. 88 mg/dl, *p*-value = 0.002, (r biserial = −0.39); 5.50% vs. 5.10%, *p*-value < 0.001, (r biserial = −0.50); 3.10 vs. 2.00 median values, *p*-value < 0.001 (r biserial = −0.50); and 137.00 mg/dl vs. 115.00 mg/dl median values (r biserial = −0.39). HDL-c values were lower in VIG compared to the NVIG (42.00 mg/dl vs. 53.50 mg/dl median values), as seen in **Table 6**.

The VIG presented higher proportion of smokers, of 46% vs. 22%, *p*-value = 0.02, of insufficiency sleep, of 84% vs. 54%, *p*-value = 0.004, respectively sedentary life habits: 89% vs. 61%, *p*-value = 0.003, with a substantial effect size (Cramer's V = 0.30).

From the family history point of view, VIG presented higher FHO and FHC, 78% vs. 50%, *p*-value = 0.007, respectively 86% vs. 59%, *p*-value = 0.005, with a substantial effect size (Cramer's V = 0.30). The results are presented in Table 3.

### 3.3 Examining relationships between vascular stiffness parameters and study variables

**Figure 3:**
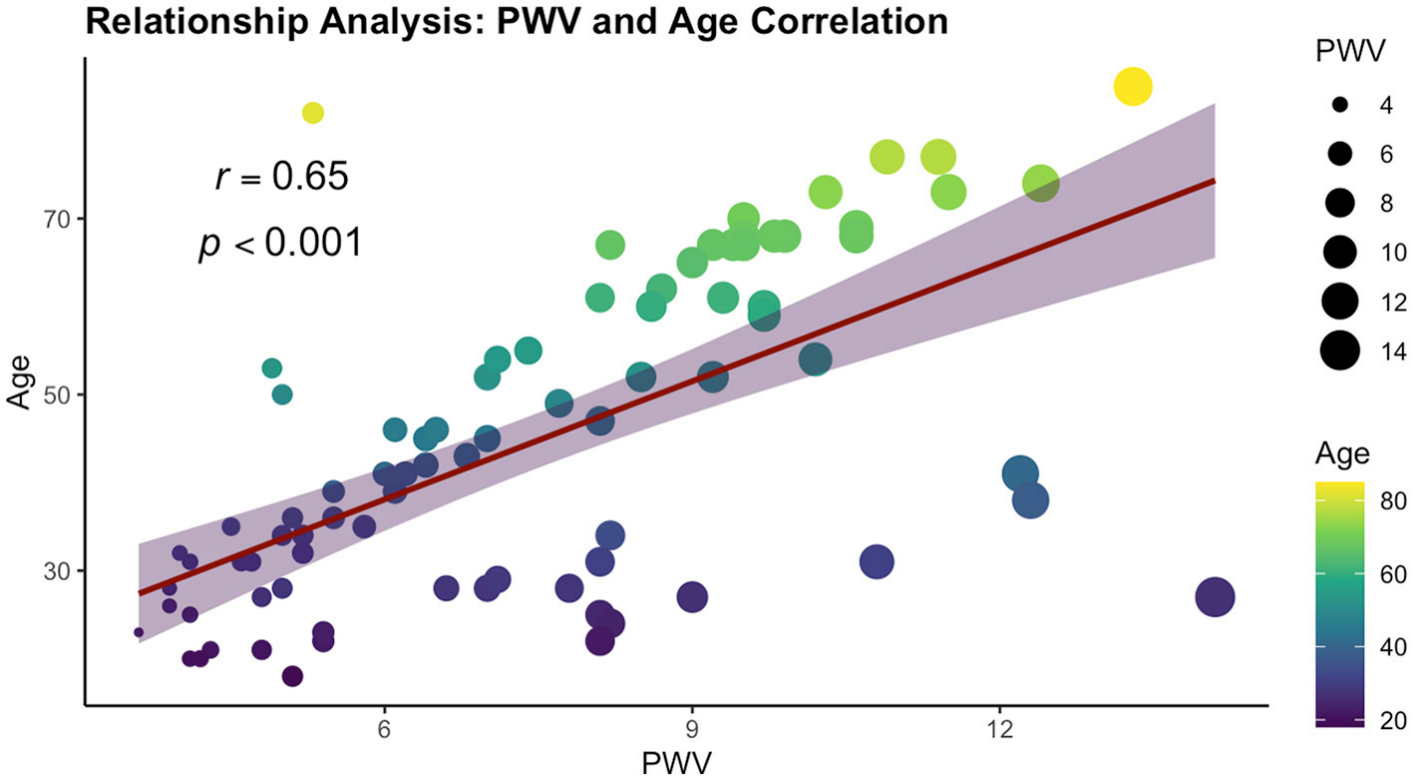
Relationship analysis: PWV and age correlation.

We performed a comprehensive correlation analysis to explore the relationships between vascular stiffness parameters and variables included in our study. From all vascular stiffness parameters examined, only PWV exhibited strong correlations with the study variables, aligning with the criteria set by Cohen's convention. Two correlations were found positive, namely age and HOMA-IR, demonstrating strong positive and statistically significant correlation (ρ = 0.65 and ρ = 0.55, respectively, *p*-value < 0.001). A statistically significant strong negative correlation was observed with HDL-c (ρ = −0.53, *p*-value < 0.001). The results are summarized in Table 4 and Figure 3.

**Table 4:** Correlation analysis between PWV and variables within study.

**Variables**	**Spearman ρ**	***p*-value**
Age	0.65	**< 0.001**
HOMA-IR	0.55	**< 0.001**
HDL-c	−0.53	**< 0.001**

HOMA-IR, Homeostatic Model Assessment of Insulin Resistance; HDL-c, High-Density Lipoprotein Cholesterol; Spearman ρ, Spearman correlation coefficient; *p*-value, Spearman correlation test. The bold values *p* < 0.05 indicates statistical significance.

We systematically investigated the relationships between vascular stiffness parameters and study variables, incorporating the stratification of patients based on BMI groups. We performed the analysis of the vascular stiffness parameters and study variables, form each category: obese, overweight and controls. In the obese subgroup, a single parameter showed a robust correlation: PWV and age (ρ = 0.50, *p*-value = 0.001).

In the overweight subgroup, both PWV and cPP exhibit correlations with the variables in the study. PWV shows significant strong positive correlations with age (ρ = 0.74, *p*-value < 0.001), HOMA-IR (ρ = 0.54, *p*-value = 0.004), and HbA1c (ρ = 0.50, *p*-value < 0.008). cPP reveals two strong positive correlations, specifically with body water (ρ = 0.50, *p*-value = 0.008) and muscle mass (ρ = 0.50, *p*-value = 0.009), respectively two strongly negative correlations with triglycerides (ρ = −0.55, *p*-value = 0.003) and TyG (ρ = −0.53, *p*-value = 0.004). The results are presented in Table 5.

**Table 5:** Vascular stiffness parameters and variables within study correlations among overweight patients.

**VRP**	**Variables**	**Spearman ρ**	***p*-value**
PWV	Age	0.74	**< 0.001**
	HOMA-IR	0.54	**0.004**
	HbA1c	0.50	**0.008**
cPP	Body Water	0.50	**0.008**
	Muscle Mass	0.50	**0.009**
	Triglycerides	−0.55	**0.003**
	TyG	−0.53	**0.004**

HOMA-IR, Homeostatic Model Assessment of Insulin Resistance; cPP, Central Pulse Pressure; PWV, Pulse Wave Velocity; TyG, Triglyceride-Glucose Index; HbA1c, Hemoglobin A1c; Spearman ρ, Spearman correlation coefficient; *p*-value, Spearman correlation test. The bold values *p* < 0.05 indicates statistical significance.

In the normal-weight patients, three vascular stiffness parameters: PWV, cSBP, and SBP showed strong correlation with the evaluated variables. Age demonstrates the most robust association with PWV across all BMI groups. Among anthropometric parameters, WC demonstrates positive correlations with all these three before mentioned arterial stiffness parameters, while BMI exhibits positive correlations with cSBP and SBP.

Total cholesterol manifests positive correlations with all three vascular stiffness variables: PWV, cSBP, and SBP. HDL-c shows positive correlations with PWV and cSBP. Additionally, a singular, notably strong and statistically significant negative correlation is observed between PWV and vitamin D values. All results are summarized in Table 6.

**Table 6:** Vascular stiffness parameters and study variables correlations among normal weight patients.

**VRP**	**Variables**	**Spearman ρ**	***p*-value**
PWV	Age	0.84	**< 0.001**
	25-OH Vit. D	−0.78	**< 0.001**
	LDL-c	0.73	**< 0.001**
	WC	0.58	**0.01**
	Total Cholesterol	0.58	**0.01**
	Triglycerides	0.56	**0.01**
	TSH	0.55	**0.02**
	Weight	0.53	**0.03**
	HDL-c	0.53	**0.02**
	Trunk Fat	0.52	**0.03**
cSBP	Uric Acid	0.65	**0.004**
	WC	0.64	**0.005**
	Total Cholesterol	0.60	**0.01**
	HOMA-IR	0.59	**0.01**
	HDL-c	−0.51	**0.03**
	BMI	0.50	**0.04**
SBP	Total Cholesterol	0.61	**0.009**
	WC	0.59	**0.01**
	Uric Acid	0.58	**0.01**
	TSH	0.54	**0.02**
	BMI	0.51	**0.03**

BMI, Body Mass Index; WC, waist circumference; HOMA-IR, Homeostatic Model Assessment of Insulin Resistance; HDL-c, High-Density Lipoprotein Cholesterol; cSBP, Central Systolic Blood Pressure; PWV, Pulse Wave Velocity; LDL-c, Low-Density Lipoprotein Cholesterol; TSH, Thyroid-Stimulating Hormone; Spearman ρ, Spearman correlation coefficient; *p*-value, Spearman correlation test. The bold values *p* < 0.05 indicates statistical significance.

### 3.4 Identifying independent predictors for PWV

To identify independent predictors of PWV, we employed both simple and multiple linear regression analyses. The initial model incorporated several predictor variables, including anthropometric factors, bioimpedance tests, cardiovascular measurements, and blood analyses. Utilizing the backward elimination method for feature selection, AIC for optimal model selection, and R^2^ adjusted for model performance assessment, we developed a predictive model. PWV increases with age, with a 0.05 increase in PWV for each year of age, female gender: with a mean value higher than the one in males with 1.71 units, alcohol consumption: 1.41 units higher scores compared with no drinkers, respectively with DBP and cSBP increase, with a 0.03 increase in PWV for every 1 mmHg change, and an increase of 0.53 units for each unit increase in the Cardiac index. Only height and body water demonstrate an inverse relationship, with a 0.06 PWV decrease for each cm, respectively 0.03 decrease in PWV for every percentage increase. Cardiac Index is positively associated, contributing to a 0.53 increase in PWV for each unit. Body Water exhibits an inverse relationship, with a 0.03 decrease in PWV for every percentage increase. HOMA-IR has a positive association, resulting in a 0.24 increase in PWV for each unit. The prediction model, consisting in the combined effect of 11 statistically significant predictors, explains 74.6% of the variance. The PWV, with R^2^ adjusted of 0.746. The results are summarized in Table 7.

**Table 7:** Independent predictors for PWV using multiple linear regression.

**Predictors**	**Estimates**	**CI**	***p*-value**
Age	0.05	0.03–0.06	**< 0.001**
Sex [M]	−1.71	−2.87–−0.56	**0.004**
Height	−0.06	−0.10–−0.01	**0.018**
Alcohol [No]	−1.41	−2.35–−0.48	**0.004**
DBP	0.03	0.01–0.06	**0.016**
cSBP	0.03	0.02–0.05	**< 0.001**
Cardiac Index	0.53	0.01–1.04	**0.044**
Body Water	−0.03	−0.05–−0.02	**< 0.001**
HOMA-IR	0.24	0.08–0.41	**0.005**
HbA1c	−0.18	−0.31–−0.05	**0.007**
HDL-c	−0.05	−0.07–−0.02	**< 0.001**
R^2^ adjusted	0.746		

DBP, Diastolic Blood Pressure; cSBP, Central Systolic Blood Pressure; HOMA-IR, Homeostatic Model Assessment of Insulin Resistance; HbA1c, Hemoglobin A1c; HDL-c, High-Density Lipoprotein Cholesterol; CI, 95% Confidence Interval. The bold values *p* < 0.05 indicates statistical significance.

### 3.5 Identifying independent risk factor for vascular impairment

To reveal the independent risk factors for vascular impairment, we employed both simple and multiple logistic regression analyses. We categorized the PWV variable into two groups: values ≤ 8 were designated for patients without vascular impairment, while values >8 were assigned to patients with vascular impairment. The backward elimination method was utilized for feature selection, AIC guided the selection of the optimal model, and Nagelkerke's R^2^ assessed the model's performance.

The regression analysis revealed that both age, cDBP (Central Diastolic Blood Pressure), and Cardiac Index serve as risk factors for the occurrence of vascular impairment. For each year increase in age, the estimated risk of developing vascular impairment rises by approximately 9%. With each unit increase in cDBP (mmHg), the estimated risk of developing vascular impairment increases by 5%, while in the case of the cardiac index, for every unit increase, the risk of developing vascular impairment rises by approximately 236%. These independent predictors collectively explain 54.1% of the variance in the 3 factors model (Nagelkerke's R^2^ = 0.541). The results are summarized in Table 8.

**Table 8:** Independent risk factors for vascular impairment using multiple logistic regression.

**Predictors**	**Estimates**	**CI**	***p*-value**
Age	1.09	1.05–1.14	**< 0.001**
cDBP	1.05	1.05–1.14	**0.034**
Cardiac Index	3.36	1.35–9.82	**0.014**
Nagelkerke's R^2^	0.541		

cDBP, Central Diastolic Blood Pressure; CI, 95% Confidence Interval. The bold values *p* < 0.05 indicates statistical significance.

### 3.6 Threshold values for age, cDBP, and cardiac index providing positive diagnostic for vascular impairment

To evaluate the efficacy of our logistic regression model, incorporating three key factors, and establish threshold values for age, cDBP, and Cardiac Index, was employed Area Under the Receiver Operating Characteristics (AUROC) statistics. The optimal cutoff point (66.03%) was pinpointed using the Youden Index (0.659), and the Confidence Interval (CI) for AUC was determined using the DeLong method.

**Figure 4:**
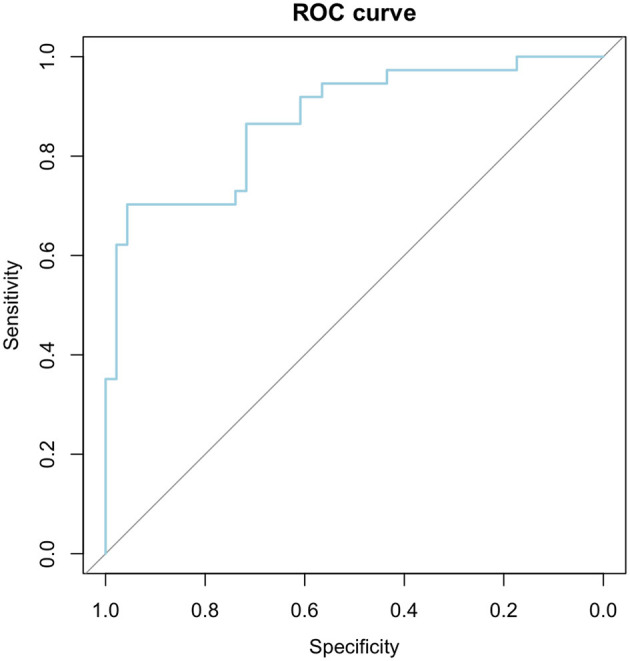
ROC curve for the 3-factors logistic regression model.

The AUROC analysis metrics facilitate a comprehensive approach of the diagnostic model, showcasing outstanding discriminative power (AUC = 0.875), with a sensitivity of 70.27%, specificity of 95.65%, and an overall accuracy of 84.33%. The identified threshold values for age, cDBP, and Cardiac Index highlight the junctures at which the model effectively distinguishes between positive and negative cases in vascular impairment diagnosis. The highly significant *p*-value (*p*-value < 0.001) reinforces the model's reliability. Detailed AUROC analysis results are summarized in Table 9 and Figure 4.

**Table 9:** AUROC analysis of the 3-factors model.

AUC (CI - DeLong)	0.875 (0.799–0.950)
Sensitivity	70.27%
Specificity	95.65%
Accuracy	84.33%
*p*-value	< 0.001
Age threshold value	32 years
cDBP threshold value	69 mmHg
Cardiac Index threshold value	2.38

AUC, Area Under the ROC curve; cDBP, Central Diastolic Blood Pressure; CI, 95% Confidence interval.

## 4 Discussion

Vascular impairment is described in overweight patients, but the phenomenon is not universal present and the magnitude of vascular damage is different in each subject. The present study assesses vascular stiffness markers utilizing the Mobil-O-Graph device in overweight individuals and examine their correlation with clinical-biological parameters and body bioimpedance parameters, in order to develop a prediction model that identifies the vulnerable patients toward vascular damage. The study not only delineats arterial stiffness parameters in individuals with obesity, but also tries to elucidate potential correlations with various biological markers beyond anthropometric measures. Consequently, valuable data were amassed and analyzed, affirming the significance of metabolic biological parameters such as LDL-c, triglycerides, TyG, HOMA-IR, and HbA1c in routine patient evaluation, given their association with non-invasive arterial stiffness. Hence, the integration of routine PWA may emerge as a feasible goal in the future for overweight adults, aiming to mitigate potential cardiovascular complications.

Atherosclerosis or the intima disease of the large and medium arteries is the main cause of arterial stiffness, which is essentially based on functional and structural changes at the vascular level. The replacement of elastic fibers with collagen, the disintegration of muscle fibers, calcium deposits and the loss of viscoelasticity are changes present at the vascular level that accompany aging (58, 59). Endothelial dysfunction is the causative factor of the appearance of atherosclerosis as a result of oxidative and nitrosative stress, inflammation, arterial hypertension and aging, and the main consequence of this physiopathological process is the narrowing of the arterial lumen (59, 60). Therefore, the increase in systolic blood pressure is a consequence of the reduction in arterial elasticity (61). Current researches indicates that arterial stiffness occurs before arterial hypertension, and not the other way around (62, 63). Therefore, the risk of cardiovascular diseases is considerably influenced by the degree of stiffness of large arteries (64, 65).

At the time of the first consultation, each subject's nutritional state as well as additional important variables were assessed from the perspective of the objective examination. These factors included age, sex, height, weight, BMI calculation, waist circumference, and waist to hip ratio. In addition to these variables, each patient benefited from a determination of body analysis by bioimpedance, in which valuable data were collected regarding the mass of adipose tissue, trunk adipose tissue muscle tissue, hydration status, fat free mass and basal metabolic rate. After data collection, the entire participant cohort was stratified based on BMI into three categories: the control group (individuals with normal weight), overweight individuals, and the obesity group. Subsequently, these data were processed in order to obtain satisfactory results with regard to the arterial stiffness parameters. Thus, for all vascular stiffness defining components, except cPP, overweight individuals obtained significantly higher values than those with normal weight. More specifically, PWV indicated statistically significant differences in nearly every connection between the nutritional status data previously presented, with the exception of sex, for which no statistically significant changes were observed for any parameter. Currently, there are convincing data that suggest a difference in arterial stiffness between the sexes, but associated with advanced age, with the level of sex hormones, anatomical data of the large vessels, and pre-existing cardiovascular pathology (66–68). So, simplifying the information, it was observed that the onset of menopause in women causes an acceleration of the increase in arterial stiffness compared to men of a similar age (68, 69). Furthermore, male gender emerged as an independent predictor for PWV values, with male participants in our study exhibiting, on average, PWV values lower by 1.71 m/s compared to females. However, this result should be interpreted cautiously due to the larger number of male subjects compared to females, in our study group.

The reduction or even the loss of elastin and collagen from the vessels is the main cause of arterial stiffness associated with aging (70). In contrast to the middle-aged population, among young adults, the role of age in the early development of arterial stiffness is uncertain, with other mechanisms such as arterial hypertension, hypercholesterolemia, inflammation, and decreased insulin sensitivity likely playing more significant roles in modifying arterial elasticity in overweight and obese individuals (24). In our study, pulse wave velocity, a critical indicator of arterial stiffness and an independent predictor of cardiovascular events (71), showed a strong and positive correlation with age across all three groups evaluated: normal weight, overweight, and obese individuals. Additionally, among the overweight group, there was another strong positive correlation with age observed cPP, a parameter known in literature and clinical practice for its independent predictability of cardiovascular events (71). Also, age was identified as an independent predictor in our research, indicating that each additional year of age leads to an increase in PWV, more precisely with each year getting older, there will be a 9% higher PWV value.

There are a multitude of risk factors that can influence the degree of vascular damage. Arterial stiffness reflects all the secondary effects of cardiovascular risk factors, starting from childhood and progressing to young adults and adulthood (52, 72, 73). Carbohydrate and lipid metabolism are important markers in the development and occurrence of arterial stiffness, the serum lipid panel markers and insulin resistance being associated with this (61, 74). A critical factor contributing to the onset of metabolic syndrome and consequent the decrease in sensitivity to insulin is the distribution of abdominal adipose tissue, which also represents a parameter that indicates the development of type 2 diabetes (75). Abdominal circumference, easily measurable in individuals with obesity, serves as a crucial parameter indicating subclinical atherosclerosis and predicting carotid intima-media thickness (76, 77). However, abdominal circumference is not the only representative parameter for insulin resistance. Elevated waist-to-hip ratio has been identified as a valuable anthropometric marker for evaluating reduced insulin sensitivity (78). BMI, a statistical measure reflecting an individual's nutritional status through weight and height, is seldom utilized as a standard for distinguishing between metabolically healthy and unhealthy obesity (79**?**). In the current research, significantly increased values of all the parameters evaluated for arterial stiffness were observed in the groups of subjects with obesity compared to overweight, respectively subject with normal weight. Similarly, when comparing overweight individuals to those with normal weight, except for cPP, all parameters exhibited statistical differences between the two groups. The percentage of individuals with low BMI, more precisely normal weight individuals, was the lowest among people without vascular damage. Also, among the normal weight group, BMI showed positive correlations only with cSBP and SBP, while abdominal circumference revealed positive correlations, in addition to cSBP and SBP, and with PWV. Despite the absence of a direct association between PWV and BMI, robust positive correlations were detected between PWV and the current body weight of each individual. Additionally, based on PWV values, significant increases in waist-to-hip ratio were noted among subjects exhibiting arterial stiffness. In their study, Kim et al. (80) similarly found no correlation between baPWV and BMI. However, they observed an association between arterial stiffness and WHR (80). Despite associations between anthropometric parameters and arterial dysfunction, BMI, WHR, and WC were not identified as unique predictors in the early onset of decreased arterial elasticity. However, height showed an inverse relationship, with each additional centimeter correlating with a decrease of 0.06 in the PWV value.

Considering the fact that the distribution of adipose tissue in the body plays an important role in predicting the risk of metabolic syndrome, insulin resistance, type 2 diabetes, but also cardiovascular events (81–83), in order to identify in detail the nutritional status of the subjects, the electrical bioimpedance analysis was used, a validated method (84) and frequently used today. Therefore, the increase in arterial stiffness can be a consequence of these unfavorable factors (85, 86). Consequently, arterial stiffness appears to exhibit a stronger correlation with abdominal adipose tissue distribution rather than overall obesity (80). Our investigation revealed a notably higher percentage of adipose tissue in the cohort exhibiting arterial stiffness impairment, particularly in the trunk region. Regarding the correlation between pulse wave velocity and parameters assessed via bioimpedance body analysis, among individuals with optimal weight, a positive association was observed between PWV and adipose tissue mass. Thus, it is anticipated that each incremental percentage rise in centrally distributed fat would correspondingly elevate PWV, the most indicative parameter of arterial stiffness in cardiovascular diseases. Furthermore, a positive correlation was noted between PWV and weight as determined by this approach. Conversely, among the overweight subgroup, significant correlations emerged between hydration status and cPP, as well as between the percentage of lean tissue (muscle mass) and cPP. Hydration status was identified as an independent predictor for PWV values, demonstrating an inverse relationship with this parameter. Thus, for every percentage increase in hydration status determined by electrical bioimpedance, a decrease in PWV of 0.03 m/s was observed.

The duration of sleep holds significant importance in predicting cardiometabolic risk. Disruption of the circadian rhythm, often attributable to nocturnal work among other factors, can induce alterations in the lipid profile (87). Consequently, sleep deprivation can lead to metabolic dysregulation and cardiovascular disorders by heightening vascular stiffness (88, 89). Moreover, individuals engaged in nocturnal employment exhibited an elevation in carotid intima-media thickness (90). Pomeroy et al. observed in adults positive associations between increased PWV values and both reduced sleep duration (below 7 h per night) and extended duration (over 9 h per night) (91). In our study, a notably higher proportion of subjects reported a sleep duration of < 7 h per night. Regarding arterial stiffness parameters, except for cDBP, all variables were significantly elevated in the group of individuals experiencing sleep deprivation compared to those with optimal sleep duration. Additionally, the percentage of individuals with sleep impairment was significantly higher in the group exhibiting vascular impairment, specifically at 84%.

Overall, data compiled from multiple studies did not reveal a clear association between alcohol consumption and arterial stiffness (27, 92, 93). Conversely, a notable predictor of PWV elevation was the total caloric intake attributable to alcohol consumption (94). Similar to any excessive behavior, alcohol consumption has been demonstrated to negatively impact quality of life, potentially leading to an increase in arterial stiffness (95). Conversely, it has been suggested that the relationship between alcohol consumption and heightened arterial stiffness may be modulated by factors such as gender or race (27). In our study, neither alcohol consumption nor sex displayed significant associations with arterial stiffness parameters. However, alcohol consumption emerged as an independent predictor for PWV. Consequently, on average, individuals who abstained from alcohol exhibited lower PWV values compared to consumers. Additionally, in the present study, abstaining from alcohol was identified as an independent predictor of arterial stiffness risk in the simple regression model.

Another significant risk factor for cardiovascular events is active tobacco smoking. The following mechanism associated with smoking are involved in the occurrence of arterial stiffness: inflammation, deterioration of endothelial function, changes in the lipid profile, acceleration of the atherothrombotic process (96). Additionally, tobacco use leads to reduced nitric oxide levels in blood vessels, promoting atherosclerosis and consequently increasing arterial stiffness (97). In our study, examination of arterial stiffness variations revealed significantly higher PWV and Aix values among smoking subjects, while the differences in other parameters were not statistically significant. Moreover, the group with increased arterial stiffness exhibited a higher proportion of smokers compared to the control group.

Regular and consistent physical activity has been shown to significantly enhance insulin sensitivity and improve body composition (98, 99), while concurrently reducing the risk of cardiovascular events (100, 101). Li et al. discovered that a longer duration of physical activity was linked to lower arterial stiffness values in patients with type 2 diabetes (98). In our study, over half of the participants in the subgroup with vascular damage reported being sedentary. Further research is warranted to precisely quantify the duration and intensity of physical activity necessary to improve arterial elasticity.

As previously discussed, the distinctiveness of the current study lies in its comprehensive analysis of both electrical bioimpedance parameters and biological markers. Just as the association between arterial stiffness and insulin resistance was elucidated from a clinical and anthropometric standpoint, significant associations were also observed from the perspective of laboratory investigations. Notably, HOMA-IR, a widely utilized index for assessing insulin resistance, relies on glucose and insulin levels obtained during fasting (102). Consequently, HOMA-IR values were markedly elevated in individuals with increased PWV. The association between HOMA-IR and PWV proved to be as significant as that of age and PWV. Among subjects with optimal weight, HOMA-IR values exhibited a positive correlation with cSBP, with observed independent predictability. Thus, for each unit increase in HOMA-IR, there was a corresponding increase in PWV by 0.24.

It's well-established that diabetes is a metabolic pathology that predisposes individuals to cardiovascular events. People with type 2 diabetes have a 2- to 4-fold increased risk of developing cardiovascular pathologies throughout their lives, including peripheral arterial disease, accidental stroke, heart failure (103, 104). However, the prevalence of arterial stiffness and cardiovascular diseases varies depending on the parameter used to define diabetes status, such as elevated HbA1c (105, 106). Elevated fasting glucose levels signify one of the initial indications of glucose-insulin metabolism dysfunction in adults (107). Elevated fasting glucose levels signify one of the initial indications of glucose-insulin metabolism dysfunction in adults (107). In the present study, individuals with elevated PWV values also exhibited higher fasting blood glucose levels. However, in other statistical analyses, no correlations were observed between these two variables. Furthermore, based on the HbA1c value, a risk predictor and indicator of long-term glycemic control (108), the subjects in our study were stratified into groups with and without diabetes to assess potential correlations with arterial stiffness. The diabetic group exhibited significantly higher arterial stiffness scores, beginning with PWV, followed by Aix, MAP, cSBP, and SBP. Conversely, HbA1c demonstrated a negative association depending on its independent predictability.

HDL-c serves as the anti-inflammatory component within the lipid panel, primarily tasked with transporting surplus cholesterol from arterial walls to hepatic cells (109). Wang et al. found, at the general population level, independent of additional risk factors such as smoking status, degree of obesity, age, an association of serum triglycerides closely correlated with arterial stiffness, while HDL-c level was not associated with this (110). On the contrary, in the China Stroke Primary Prevention Trial, a link was found between arterial stiffness and lipid profile parameters among the rural Chinese population that associated essential hypertension (111). We also found that the low value of HDL-c is an independent predictor for PWV. In our study, there was a statistically significant negative association observed between HDL-c low levels and PWV. On the other hand, in the control group, positive correlations between HDL-c and cSBP were identified. Therefore, there was an association between increased HDL-c levels and a 0.05 m/s decrease in PWV.

There is no direct relationship between LDL-c levels and markers of arterial stiffness. Various risk factors such as age, concurrent pathologies like diabetes and hypertension, as well as nutritional status, might influence the impact of LDL-c on PWV (61). Kilic et al. reported an association between the two parameters, identifying that the group of patients who had the highest PWV value also had increased LDL-c values (112). Also in the same direction, Zhao et al. found a significant association between LDL-c and baPWV values, but additionally this correlation was not identified among women (113). On the contrary, Wen et al. did not identify an association between increased values of LDL-c and a-PWV (114). A similar result was identified in the case of another study, in which it was observed that the LDL-c value could not independently predict arterial stiffness (115). In our study, the results were similar, with an increased value of LDL-c in group with high arterial stiffness.

The TyG index is a parameter derived from fasting blood glucose and triglyceride levels determined after a minimum 8-h fast (116). It has been proposed as a measure for assessing insulin resistance, with its validity compared to the euglycemic-hyperinsulinemic clamp, considered the “gold standard” for insulin resistance assessment (102, 117). Several studies have compared TyG with HOMA-IR, consistently demonstrating superior results with TyG, irrespective of the presence or absence of diabetes (117, 118). Recent research has also supported a positive association between TyG levels and arterial stiffness, both in healthy populations and individuals with arterial hypertension (119, 120). In our study, among overweight patients, cPP exhibited a negative and statistically significant correlation with TyG.

Even though vitamin D deficiency is relatively common among the population (121), our current research revealed lower values of vitamin D associated with increasing PWV, respectively arterial stiffness. Within the control group, we identified only one strong and statistically significant negative correlation between PWV and 25-OH-Vitamin D. The role of vitamin D in arterial stiffness hinges on its control over the interaction between vitamin D ligand and the vitamin D receptors at the endothelial level (122).

It has been noted that elevated levels of TSH, whether in subclinical or manifest hypothyroidism, are linked to increased PWV values, indicative of arterial stiffness (123, 124). However, in our current study, no associations were observed between TSH and arterial stiffness parameters among subjects with overweight and obesity. Nonetheless, positive correlations were identified between TSH levels and both SBP and PWV within the control group.

The final product of purine metabolism in humans, serum uric acid, has been strongly linked to various metabolic pathologies, including type 2 diabetes, gout, and changes in lipid profiles (125–127). Tiam et al. demonstrated a positive correlation between serum uric acid levels and the risk of arterial stiffness, particularly among women, with further evidence of progression (128).

In addition to identifying the possible associations between anthropometric parameters, body analysis by bioimpedance and laboratory analyzes with the PWV value, implicitly with arterial stiffness in overweight and obese people, the particularity of our research aimed to construct a predictive model for individual risk factors affecting PWV and vascular damage. Independent predictors for PWV in multiple regression analysis included age, male gender, lifestyle factors (abstinence from alcohol), anthropometric variables (height), bioimpedance analysis parameters (hydration status), and laboratory markers such as HOMA-IR, HbA1c, and HDL-c. Furthermore, logistic regression models were employed to assess risk factors for vascular damage, revealing age, cDBP, and cardiac index as significant variables. Each year of aging increased the estimated risk of developing arterial stiffness by 9%, while each unit increase in cDBP and cardiac index elevated the risk of vascular insufficiency by 5% and approximately 236%, respectively. Mobil-O-Graph measurements facilitated the determination of cDBP and cardiac index. The independent prediction model highlighted threshold values for these parameters, aiding in the diagnosis of arterial stiffness, with an AUROC analysis demonstrating a specificity of 95.65%, sensitivity of 70.27%, and overall accuracy of 84.33%.

The present research successfully validated the primary hypothesis, affirming that abdominal obesity contributes significantly to early arterial stiffness in adults. Consequently, the consideration of these parameters is crucial during routine assessments, particularly among overweight individuals. Furthermore, our research yielded additional insights by identifying various correlations between arterial stiffness markers and anthropometric parameters, laboratory investigations, and electrical bioimpedance—a technique employed to quantify adipose tissue mass. Additionally, independent risk factor models were developed for both PWV values and endothelial damage, yielding remarkable outcomes. Despite promising results, there are several limitations associated with PWV determination. Firstly, the small participant sample size serves as the primary constraint of our study. Secondly, the execution of PWV requires strict adherence to procedural guidelines, including measurements taken under appropriate conditions following a 10-min rest period with the patient in a supine position. Factors such as heart rate and blood pressure must also be taken into account when interpreting PWV results, as they can influence the outcome (129). Moreover, the uncertainty surrounding ultrasonographic measurements of flow-mediated dilation as a standard for early monitoring of arterial wall changes presents another limitation in our study (130).

## 5 Conclusions

In adults, particularly those with centrally distributed obesity, there is a progressive decline in the elasticity of large blood vessels, resulting in heightened arterial stiffness. Anthropometric measures such as abdominal circumference, BMI, and WHR have been correlated with increased PWV. Alterations in lipid profiles, decreased levels of 25-OH-Vitamin D, the HOMA-IR index, glycemic levels in young individuals, and HbA1c contribute similarly to the progression of arterial stiffness. In healthy individuals without endocrinological pathologies, TSH levels were not found to be significantly associated with arterial stiffness. Additionally, notable associations were observed in body bioimpedance analysis markers, particularly regarding adipose tissue mass at the trunk level and PWV. In summary, assessing and monitoring PWV at initial consultations and throughout the progression of overweight or obese individuals, as well as those at risk of weight gain despite being of normal weight, offers a reliable, non-invasive, and relatively simple method that yields valuable medical insights. Consequently, evaluating arterial stiffness can be deemed a dependable approach to preventing cardiovascular events linked to obesity and facilitating the comprehensive management of such pathologies.

## Data Availability

The original contributions presented in the study are included in the article/Supplementary material, further inquiries can be directed to the corresponding author.
