# Supplementary material for: Development of a comprehensive risk prediction model for arterial stiffness assessment in individuals with obesity
Source: Front Med (Lausanne). 2024 Aug 19;11:1430437. doi: 10.3389/fmed.2024.1430437 (PMC11368134; doi:10.3389/fmed.2024.1430437)
Supplement: Supplementary file 1 [file Table_1.docx]

Supplementary Material

# Supplementary Tables

**Supplementary Table 1 - Continuous variables within the study**

| N = 83 | Variable | Normal weight | Overweight | Obesity | *p-value* |
| --- | --- | --- | --- | --- | --- |
| Anthropometric measurements | **Age *(years)*** | 34.00 | 43.50 | 45.00 | 0.38 |
|  | ***Height *(cm)*** | 165.53 | 165.31 | 165.95 | 0.96 |
|  | ***Weight *(kg)*** | 56.91 | 73.67 | 97.63 | **<0.001** |
|  | ***WC *(cm)*** | 71.47 | 92.88 | 114.90 | **<0.001** |
|  | **WHR** | 0.78 | 0.88 | 0.99 | **<0.001** |
| Cardiovascular assessments | **SBP *(mmHg)*** | 103.00 | 119.50 | 128.50 | **<0.001** |
|  | ***DBP *(mmHg)*** | 65.41 | 71.65 | 77.05 | **<0.001** |
|  | ***MAP *(mmHg)*** | 83.88 | 94.92 | 103.03 | **<0.001** |
|  | **HR *(b/min)*** | 72.00 | 76.00 | 76.00 | 0.55 |
|  | **cSBP *(mmHg)*** | 107.00 | 121.50 | 127.00 | **<0.001** |
|  | ***cDBP *(mmHg)*** | 68.18 | 72.58 | 78.65 | **0.006** |
|  | **cPP *(mmHg)*** | 42.00 | 49.00 | 50.00 | 0.17 |
|  | **PWV *(m/s)*** | 4.80 | 8.40 | 7.75 | **<0.001** |
|  | **AIx *(%)*** | 10.00 | 23.50 | 33.00 | **<0.001** |
|  | **Cardiac Index *(L/min/***$\boldsymbol{m}^{\boldsymbol{2}}$***)*** | 2.60 | 2.20 - 3.10 | 0.02 | 0.34 |
| Bioimpedance tests | **Fat Mass *(%)*** | 20.30 | 36.35 | 45.20 | **<0.001** |
|  | **Fat-Free Mass *(kg)*** | 45.30 | 44.35 | 53.15 | **<0.001** |
|  | **Trunk Fat *(%)*** | 17.40 | 35.05 | 44.45 | **<0.001** |
|  | **Muscle Mass *(%)*** | 75.60 | 59.95 | 52.00 | **<0.001** |
|  | **BMR *(kcal/day)*** | 1344.00 | 1398.00 | 1632.00 | **0.002** |
|  | **Body Water *(%)*** | 58.80 | 46.25 | 40.75 | **<0.001** |
|  | **Impedance *(***$\boldsymbol{\Omega}$***)*** | 655.00 | 644.00 | 541.50 | **<0.001** |
| Blood analyses | **Fasting glucose *(mg/dL)*** | 81.00 | 95.50 | 96.50 | **<0.001** |
|  | **HbA1c *(%)*** | 4.80 | 5.30 | 5.55 | **<0.001** |
|  | **HOMA-IR** | 1.00 | 2.35 | 3.17 | **<0.001** |
|  | ***Total Cholesterol *(mg/dL)*** | 132.29 | 179.73 | 208.15 | **<0.001** |
|  | ***LDLc *(mg/dL)*** | 69.37 | 132.88 | 141.10 | **<0.001** |
|  | **HDLc *(mg/dL)*** | 64.00 | 45.50 | 47.50 | **<0.001** |
|  | **Triglycerides *(mg/dL)*** | 100.00 | 98.50 | 147.00 | **0.002** |
|  | ***Uric Acid *(mg/dL)*** | 5.08 | 5.50 | 5.53 | 0.28 |
|  | **25-OH Vit. D *(nmol/L)*** | 25.10 | 22.80 | 21.00 | **0.006** |
|  | **TyG** | 3900.00 | 4416.75 | 6517.25 | **0.001** |
|  | **TSH *(ng/dL)*** | 1.30 | 1.95 | 3.25 | **0.001** |
|  | ***FT4 *(ng/dL)*** | 12.20 | 11.50 | 9.87 | **0.04** |

Abbreviations: N - number of participants; BMI - Body Mass Index; WC - waist circumference; WHR - Waist-to-Hip Ratio; SBP - Systolic Blood Pressure; DBP - Diastolic Blood Pressure; MAP - Mean Arterial Pressure; HR - Heart Rate; cSBP - Central Systolic Blood Pressure; cDBP - Central Diastolic Blood Pressure; cPP - Central Pulse Pressure; PWV - Pulse Wave Velocity; AIx - Aortic Augmentation Index; BMR - Basal Metabolic Rate; HbA1c - Hemoglobin A1c; HOMA-IR - Homeostatic Model Assessment of Insulin Resistance; LDLc - Low-Density Lipoprotein Cholesterol; HDLc - High-Density Lipoprotein Cholesterol; TyG - Triglyceride-Glucose Index; TSH - Thyroid-Stimulating Hormone; FT4 - Free Thyroxine. *p*-value derived from the ANOVA test for normally distributed data and for Kruskal-Wallis for non-normally distributed data; * - variables with normal distribution.
