# Supplementary material for: Development of a comprehensive risk prediction model for arterial stiffness assessment in individuals with obesity
Source: Front Med (Lausanne). 2024 Aug 19;11:1430437. doi: 10.3389/fmed.2024.1430437 (PMC11368134; doi:10.3389/fmed.2024.1430437)
Supplement: Supplementary file 2 [file Table_2.docx]

Supplementary Material

# Supplementary Tables

**Supplementary Table 2. Differences in Vascular Impairment groups across study variables**

| Variables | Mann-Whitney U *p*-value | 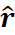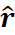 biserial |
| --- | --- | --- |
| Age | **<0.001** | -0.61 |
| Height | 0.08 | 0.22 |
| Weight | 0.31 | -0.13 |
| BMI | 0.16 | -0.18 |
| WC | 0.05 | -0.25 |
| WHR | **0.02** | -0.31 |
| HR | **0.01** | 0.32 |
| Cardiac Index | **0.001** | -0.41 |
| Fat Mass | **0.02** | -0.29 |
| Fat-Free Mass | 0.49 | 0.09 |
| Trunk Fat | **0.04** | -0.26 |
| Muscle Mass | **0.04** | 0.26 |
| BMR | 0.94 | 0.009 |
| Body Water | 0.13 | 0.20 |
| Impedance | **0.01** | 0.33 |
| Fasting glucose | **0.002** | -0.39 |
| HbA1c | <**0.001** | -0.50 |
| HOMA-IR | **<0.001** | -0.50 |
| Total Cholesterol | 0.11 | -0.20 |
| LDLc | **0.007** | -0.34 |
| HDLc | **<0.001** | 0.49 |
| Triglycerides | 0.67 | -0.06 |
| Uric Acid | 0.60 | -0.07 |
| 25-OH Vit. D | **0.002** | 0.39 |
| TyG | 0.30 | -0.13 |
| TSH | 0.05 | -0.26 |
| FT4 | 0.24 | -0.15 |

Abbreviations: BMI - Body Mass Index; WC - waist circumference; WHR - Waist-to-Hip Ratio; SBP - Systolic Blood Pressure; DBP - Diastolic Blood Pressure; MAP - Mean Arterial Pressure; HR - Heart Rate; cSBP - Central Systolic Blood Pressure; cDBP - Central Diastolic Blood Pressure; cPP - Central Pulse Pressure; PWV - Pulse Wave Velocity; AIx - Augmentation Index; BMR - Basal Metabolic Rate; HbA1c - Hemoglobin A1c; HOMA-IR - Homeostatic Model Assessment of Insulin Resistance; LDLc - Low-Density Lipoprotein Cholesterol; HDLc - High-Density Lipoprotein Cholesterol; TyG - Triglyceride-Glucose Index; TSH - Thyroid-Stimulating Hormone; FT4 - Free Thyroxine; .
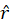

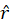
 biserial- measures the magnitude of the difference with Mann-Whitney U test.
